# MicroRNA-138 is a Prognostic Biomarker for Triple-Negative Breast Cancer and Promotes Tumorigenesis via TUSC2 repression

**DOI:** 10.1038/s41598-019-49155-4

**Published:** 2019-09-03

**Authors:** Srikanth Nama, Manish Muhuri, Federica Di Pascale, Shan Quah, Luay Aswad, Melissa Fullwood, Prabha Sampath

**Affiliations:** 1 Skin Research Institute of Singapore, Agency for Science Technology & Research (A*STAR), Singapore, 138648 Singapore; 2 Horae Gene Therapy Center, University of Massachusetts Medical School, Worcester, MA 01605 USA; 3 Department of Biochemistry, Yong Loo Lin School of Medicine, National University of Singapore, Singapore, Singapore; 4 Program in Cancer and Stem Cell Biology, Duke-NUS Medical School, 8 College Road, Singapore, 169857 Singapore; 5 Cancer Science Institute of Singapore, National University of Singapore, Singapore, Singapore; 6 School of Biological Sciences, Nanyang Technological University, Singapore, Singapore

**Keywords:** Breast cancer, miRNAs

## Abstract

Breast cancer manifests as a spectrum of subtypes with distinct molecular signatures, and different responses to treatment. Of these subtypes, triple-negative breast cancer (TNBC) has the worst prognoses and limited therapeutic options. Here we report aberrant expression of microRNA-138 (miR-138) in TNBC. Increased miR-138 expression is highly specific to this subtype, correlates with poor prognosis in patients, and is functionally relevant to cancer progression. Our findings establish miR-138 as a specific diagnostic and prognostic biomarker for TNBC. OncomiR-138 is pro-survival; sequence-specific miR-138 inhibition blocks proliferation, promotes apoptosis and inhibits tumour growth *in-vivo*. miR-138 directly targets a suite of pro-apoptotic and tumour suppressive genes, including tumour suppressor candidate 2 (TUSC2). miR-138 silences *TUSC2* by binding to a unique 5′-UTR target-site, which overlaps with the translation start-site of the transcript. Over-expression of TUSC2 mimics the phenotype of miR-138 knockdown and functional rescue experiments confirm that TUSC2 is a direct downstream target of miR-138. Our report of miR-138 as an oncogenic driver in TNBC, positions it as a viable target for oligonucleotide therapeutics and we envision the potential value of using antimiR-138 as an adjuvant therapy to alleviate this therapeutically intractable cancer.

## Introduction

Breast cancer is the most common cancer in women (~12% of new cancer diagnoses, 25% of cancers in women;)^1^, and a leading cause of female cancer-related death worldwide (<500,000 deaths per year)^2^. Clinical difficulties in managing breast cancer result in part from the heterogenous nature of the disease – breast tumours fall into a spectrum of subtypes with distinct morphologies, molecular features, and responses to treatment.

Breast tumours may be histologically classified based on their expression of the oestrogen and progesterone hormone receptors (ER and PR respectively), and human epidermal growth factor receptor 2 (HER2). The ‘luminal’ subtypes (Luminal A and Luminal B) express ER. This ER + phenotype generally produces good clinical outcomes due to the availability of effective strategies targeting signalling through the oestrogen receptor^3^. Similarly, treatment regimes targeted at the HER2 pathway have revolutionized the treatment of HER2 + breast carcinomas^4^. In contrast, triple-negative breast tumours do not express ER, PR, or HER2. Chemotherapy is the mainstay for management of triple-negative breast cancer (TNBC) due to the lack of targeted therapeutics. The need for tailored treatment regimes for TNBC is reflected in its poorer clinical outcomes relative to other breast cancers^5^. Triple-negative tumours represent ~20% of breast cancer diagnoses. They tend to occur in younger patients (<50 years), progress rapidly, and are more likely to metastasize to the brain and viscera relative to HER2 + cancers^5^. As a result, TNBC patients are significantly less likely to survive than other breast cancer patients following the first metastatic event.

At this juncture, it is important to note the subtle distinction between TNBC and basal-like breast carcinomas. Both subtypes show similarities in their clinical progression, including early age of onset and aggressive progression. Considerable overlap (>70%) exists between basal-like and triple-negative cancers^3^, but the two subtypes are not synonymous. Sub-classification of TNBCs from basal-like tumours is necessary – the ‘TNBC’ designation concerns hormone receptor and HER2 status, which have direct clinical relevance. In contrast, the ‘basal-like’ classification is usually based on immunohistochemical staining for the expression of epidermal growth factor receptor (EGFR) and cytokeratins (especially CK5/6)^6,7^, the clinical utility of which is less straightforward. The large extent of overlap between the two subtypes occurs because most basal-like tumours also lack oestrogen receptor (ER), progesterone receptor (PR) and HER2 expression. A five marker panel (ER-PR-HER2–EGFR-CK5/6) allows sub-classification of TNBCs as basal-like (or Core Basal, CB) when EGFR and/or CK5/6 are positive or five negative (5NP) if all markers are negative^8^. In this report, we will focus on TNBCs regardless of basal-like status in order to address the lack of targeted therapeutics in ER-/PR-/HER2- triple-negative tumours,

Targeting microRNAs (miRNAs), or their associated regulatory networks, which are dysregulated in TNBC might represent a viable strategy for targeted therapeutics. miRNAs are short (18–23 nt) non-coding RNAs that regulate virtually all biological functions via post-transcriptional gene silencing. Altered miRNA expression is common during cancer initiation and metastasis^6^. A systems level analysis of miRNA expression in human breast tumours revealed that specific miRNAs may serve as potential oncogenes or tumour suppressors and function by modulating the immune response that characterizes these tumours^9^. In fact, miRNA expression signatures are also correlated with the hormone receptor status in breast cancer. Three classes of miRNA signatures corresponding with ER (miR-342, miR-299, miR-217, miR-190, miR-135b and miR-218), PR (miR-520g, miR-377, miR-527–518a and miR-520f-520c) and HER2 (miR-520d, miR-181c, miR-302c, miR-376b and miR-30e) have been characterized, respectively^10^. Moreover, as many as 133 miRNAs are differentially expressed between tumours and healthy tissue^7^. An array of studies has been published on miRNA signatures in TNBC that have elucidated the roles of miRNAs in the progression or suppression of tumors. A number of them are overexpressed, namely miR-221, miR-222, miR-100, miR-146a, miR-125b^11–14^, miR-29a, miR-31, miR-130a, miR-140-3p, miR-455, miR-199a/b-3p^14^, miR-135-5p, miR-18-5p, miR-9-5p, miR-522-3p^15^, while miRNA genes like miR-26a^16^, miR-20a-5p^17^, miR-124^18^, miR-200, miR-182, miR-141, miR-375, miR-203^11–13^, miR-190-5p, miR-449a^15^, have reduced expression in TNBC tumours. Among this list of differentially expressed miRNAs, we have previously demonstrated oncogenic potential for miR-138 in recurrent malignant gliomas, and were interested in exploring its roles in the context of breast cancer. In gliomas, miR-138 is transcribed by RNA Pol III^8^ and promotes the survival of glioma stem cells^9^. Aberrant miR-138 expression has been reported in various cancers including anaplastic thyroid carcinoma (ATC), NSCLC, and gallbladder carcinoma^10–12^. Increased expression of miR-138 in TNBC has been reported^11,13,19,20^. miR-138 may modulate metastasis and epithelial-mesenchymal transformation (EMT)^21^. By and large, epigenetic and genetic changes in cancer cells during malignant transformation alter the suite of mRNAs available as targets for miRNAs. Therefore, the cellular context-specific function of miRNAs depends on the available cohorts of downstream effectors^13^. Hence, we hypothesize that in breast cancer, tumour cells might differ in their behaviour, including their expression and utilization of miR-138, by subtype.

In this report, we present our finding that miR-138 is a specific molecular signature of triple-negative breast cancers. It is expressed in cells and tissues derived from triple-negative carcinomas and absent in both luminal and HER2 + breast cancers. This expression of miR-138 is clinically significant in TNBC and we demonstrate that miR-138 is a prognostic biomarker for breast cancer pathogenesis. We also identify TUSC2, a tumour suppressor, as one of the direct targets of miR-138. *In vivo* tumour formation is inhibited by miR-138 knockdown, suggesting that targeted therapy may unlock new strategies for the management of triple-negative breast cancers with improved patient outcome.

## Results

### MicroRNA-138 is a diagnostic biomarker for triple-negative breast cancer

**Figure 1:**
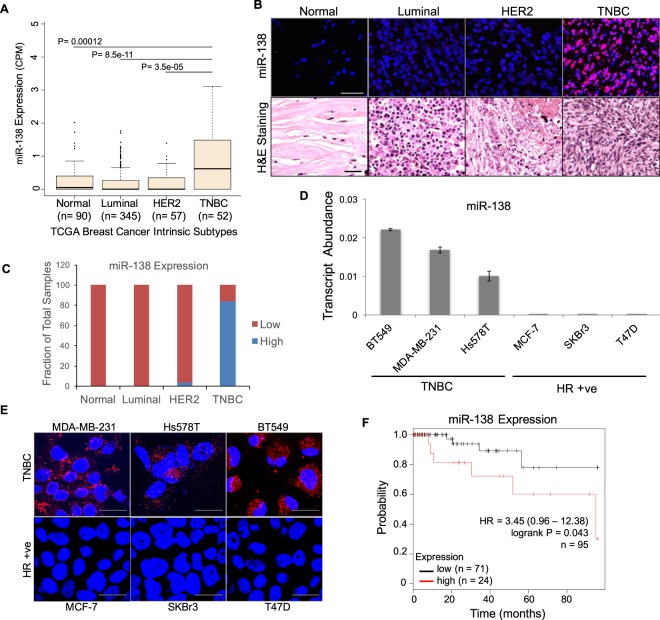
MicroRNA-138 is a potential diagnostic biomarker for triple-negative breast cancer: (**A**) miR-138 expression data sourced from the TCGA database, represented as box plots in breast cancer subtypes along with normal tissue samples. Note that TNBC shows highest mean expression of miR-138. (**B**) *In situ* hybridization with LNA probes specific for mature miR-138 on patient tissue sections from tissue array (upper panel) [normal (n = 6), luminal (n = 51), HER2 (n = 33) and TNBC (n = 54). miR-138 is stained in red and nuclei are stained blue. Scale bar_,_ 50 µm. Hematoxylin & Eosin staining for the same (lower panel) Scale bar - 100 µm. (**C**) Bar graph represents quantification of miR-138 expression in tissue samples from indicated groups. Note that a substantial proportion (84%) of TNBC sections express high levels of miR-138 compared to other subtypes or normal tissue sections. (**D**) Bar graph representing transcript abundance of mature miR-138 in indicated breast cancer cell lines. Note that TNBC cell lines express elevated levels of miR-138 compared to HR + ve cell lines. Error bars represent the standard deviation. (**E**) Expression of miR-138 detected by *in situ* hybridization in TNBC (upper panel) or HR + ve cell lines (lower panel). miR-138 is stained in red and nuclei are stained blue. Scale bar, 20 µm. (**F**) Kaplan-Meier survival curves with statistical significance by log-rank test of two groups representing TNBC subtype (n = 95). Elevated expression of miR-138 correlates with increased mortality.

A screen from 544 breast cancer patients in The Cancer Genome Atlas (TCGA) database revealed that triple-negative breast tumours express significantly higher levels of miR-138 than luminal tumours, HER2 + tumours, or healthy breast tissue (TNBC: n = 52, luminal: n = 345, TNBC vs luminal p < 0.0001, Fischer’s exact test; Fig. 1A). To further validate this finding, we screened patient tissue array samples using fluorescent *in situ* hybridization (FISH) for miR-138. Elevated miR-138 expression was noted in 84% (n = 43/51) of the TNBC tumour sections, while little or no miR-138 was seen in luminal (n = 54) and majority of the HER2 tumour sections (n = 33) (Fig. 1B,C).

Further, our findings were validated by specific expression of miR-138 in TNBC cell lines. Using stem loop qRT-PCR, we observed that three cell lines derived from TNBC tumours (MDA-MB-231, Hs578T, and BT549) expressed high levels of miR-138. In contrast, human breast epithelial cell line (MCF10A) or cell lines derived from hormone receptor positive (HR + ve) tumours (MCF7, SKBR3, and T47D) showed little or no miR-138 expression (Figs S1A, 1D). We confirmed these findings by *in situ* hybridization (ISH), using specific probes to detect miR-138 (Fig. 1E). Our ISH data was consistent with our qRT-PCR data, validating significant miR-138 expression only in TNBC cell lines.

To understand the clinical relevance of this TNBC-specific miR-138 expression, we analyzed miR-138 expression in TCGA database in correlation with patient survival. In an unsorted TCGA dataset containing 579 breast cancer patients, we noted no significant association between miR-138 expression and patient outcome (Hazard Ratio = 1.04 (0.61–1.78), n = 579, *P* = 0.87) (Fig. S1B). Filtering this dataset and restricting this analysis to 95 TNBC patients reveals a significant correlation between miR-138 level and patient prognosis. Increased miR-138 expression is associated with increased mortality (Hazard Ratio = 3.45 (0.96–12.38), n = 95, *P* = 0.043; (Fig. 1F). Our data suggests that miR-138 distinguishes TNBC tumours from other breast cancer subtypes and serves as a specific biomarker for this subtype. We also note that miR-138 is a prognostic marker with reference to TNBC progression.

### MicroRNA-138 is a pro-survival oncomiR for triple-negative breast cancer

**Figure 2:**
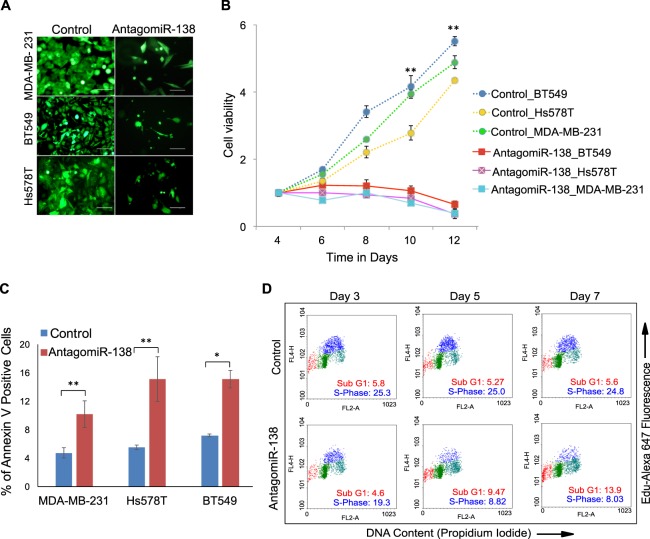
MicroRNA-138 is a pro-survival oncomiR for TNBC subtype: (**A**) Fluorescent images of TNBC cell lines transduced with indicated lentivirus expressing antagomiR-138 or scrambled control. (**B**) The line graph indicates that the number of viable cells increased with time in cells transduced with scrambled control, but not in cells transduced with antagomiR-138. Note the results are consistent in all three cell lines. (**C**) Bar graph depicts percentage of Annexin V positive cells in TNBC cell lines transduced with indicated lentivirus. (**D**) Dual colour cell-cycle analysis of MDA-MB-231 cells transduced either with scramble or antagomiR-138 at indicated time points. Note the decrease in S-phase (blue) cell population and concomitant increase in subG1 (red) population in antagomiR-138 transduced cells compared to scrambled control.

To study the consequences of aberrant miR-138 expression in TNBC, we generated lentiviral constructs encoding antagomiRs against miR-138. These were used to create stable miR-138 knockdowns in TNBC cell lines: MDA-MB-231, Hs578T, and BT549. Corresponding transductions were performed, incorporating a non-targeting scrambled construct, to control for off-target effects. Knockdown efficiency was verified by qRT-PCR (Fig. S2A). TNBC cells expressing lentiviral-encoded antagomiR-138 failed to grow and a significant reduction in cell number was apparent (Fig. 2A). This effect was not seen in HR + ve cell lines transduced with the same construct (Fig. S2B). No growth retardation was observed with the scrambled control construct in any of the cell lines tested (Figs 2A and S2B). The effect of antagomiR-138 expression on cell viability was assayed over time in MDA-MB-231, Hs578T, and BT549 cells (Fig. 2B). In contrast to the scrambled controls, where cell viability increases substantially over 12 days, miR-138 knockdown decreases cell viability over time. The inhibitory effects of miR-138 knockdown on cell viability are also apparent in 3D organoid culture. AntagomiR-138 expression in MDA-MB-231 and BT549 cells significantly reduced colony formation in an anchorage-independent soft agar assay, relative to the scrambled control (Figs S2C and S2D).

The above effects of miR-138 on promoting cell viability might result from reduced apoptosis, increased cell proliferation, or a combination of both processes. We assayed cells for Annexin V, an early apoptotic marker, to test for an effect of miR-138 on apoptotic inhibition. AntagomiR-138 transduction increased the fraction of Annexin V-positive cells relative to that in the scrambled control (Fig. 2C). To confirm the role of miR-138, we further analyzed cellular DNA content using a dual-colour flow cytometric analysis in MDA-MB-231 cells transduced with either antagomiR-138 relative to that in the scrambled control. In the presence of the antagomiR, cellular entry into S-phase was reduced (Fig. 2D). Additional observations such as an increase in the sub-G1 population (Fig. 2D), suggests the cells may be undergoing apoptotic cell death in the presence of the antagomiR.

### Depletion of miR-138 leads to apoptotic cell death *in vitro* and prevents tumorigenesis *in vivo*

**Figure 3:**
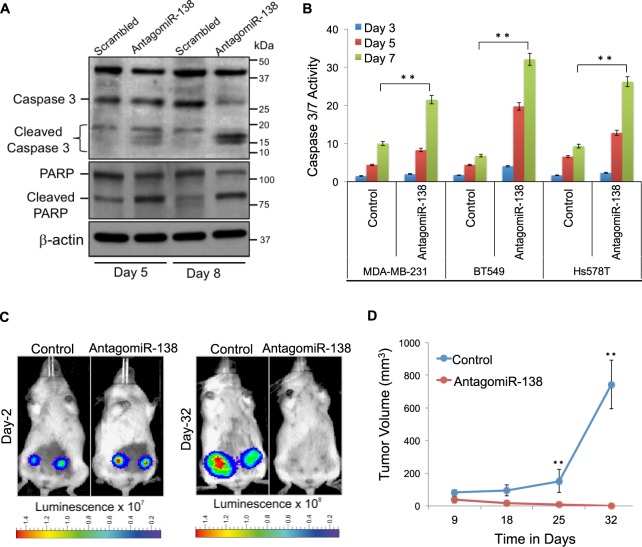
Depletion of miR-138 leads to apoptotic cell death *in vitro* and prevents tumorigenesis *in vivo*: (**A**) Western blot analysis for cleaved caspase 3, and cleaved PARP; β-Actin serves as a loading control. (**B**) Caspase-3/7 activity, normalized to the cell number, increased in antagomiR-138 transduced cells relative to the scramble control. **C**) Representative bioluminescent images of NSG mice implanted with antagomiR-138 or scrambled control expressing MDA-MB-231 cells, at indicated time points post-engraftment in mammary fat pads. (**D**) Line graph represents real time analysis of tumour volume in mice (n = 10). Error bars represent the standard deviation (Student’s *t*-test; *[P < 0.05], **[P < 0.001]).

Detection of 89 kDa cleaved PARP, and presence of 19 kDa cleaved caspase upon depletion of miR-138 (Fig. 3A), indicates enhanced apoptosis. Finally, compared to the scrambled control we observed an increase in caspase-3/7 activity in response to miR-138 knockdown confirming that depletion of miR-138 enhances apoptosis in TNBC cells,. This effect is consistent in MDA-MB-231, Hs578T, and BT549 (Fig. 3B). These data demonstrate that miR-138 promotes viability in triple-negative tumour cells by enhancing proliferation and simultaneously suppressing apoptosis *in vitro*.

Having established that miR-138 supports cell survival in culture, we sought to study *in vivo* effects of miR-138 on tumourigenesis. Luciferase-expressing MDA-MB-231 cells, transduced with either antagomiR-138 or the scrambled control, were implanted into the mammary fat pad in immunocompromised mice (NOD.*Cg-Prkdc*^*scid*^*Il2rg*^*tm1Wjl*^/SzJ mice; n = 10). Both the antagomiR-expressing and control cells successfully establish tumours following engraftment (Fig. 3C). After 32 days, control tumours attain significantly larger sizes compared to those established using miR-138 knockdown cells (0.57 mm^3^ for antagomiR-138 compared to 740.9 mm^3^ for control, p = 0.0082; Fig. 3D and S3A). To validate our observation that miR-138 knockdown reduces cell proliferation, we stained xenograft tumour sections for Ki-67, which is a marker for proliferation. Ki-67 staining was notably more abundant in control tumour sections compared to those obtained under miR-138 knockdown conditions (Fig. S3B). We also performed immunohistochemical staining on these sections for the apoptotic marker CASP3 (Fig. S3C). CASP3-expressing cells were observed in miR-138 knockdown tumour sections, albeit at a lower frequency than Annexin V-positive cells in 2D culture (Fig. 2C).

Together, these findings support a role for miR-138 in augmenting the growth and survival of triple-negative breast cancer cells. Our results are consistent both in cell culture as well as in xenograft mouse models. They lend support to our hypothesis that miR-138 has a pro-survival function in the TNBC subtype.

### Tumour suppressor gene TUSC2 is a direct target of miR-138

**Figure 4:**
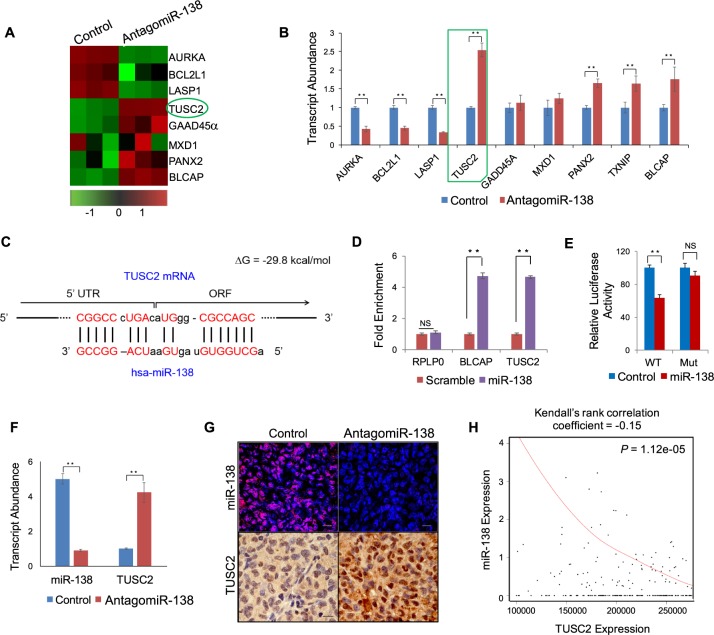
Tumour suppressor gene TUSC2 is a direct target of miR-138: (**A**) Heat map representing expression values of selected genes from microarray data analysis in MDA-MB-231 cells (scrambled control vs antagomiR-138). Red and green shades indicate deviation relative to the individual mean values of the genes. (**B**) qRT-PCR validation of selected miR-138 target genes from microarray. Note that TUSC2 showed highest expression levels upon miR-138 knockdown. (**C**) Binding site of miR-138 at 5′ UTR region of TUSC2 mRNA, complementary nucleotides are shown in red. (**D**) Bar graph represents qRT-PCR analysis of enriched transcripts by RNA immuno-precipitation (RIP). Note that TUSC2 mRNA is enriched by biotinylated miR-138 oligos in HEK293T cells, RPLP0 serves as a negative control and BLCAP serves as the positive control. (**E**) Functional validation of miR-138 and TUSC2 interaction by chimeric luciferase assay. Bar graph represents normalized luciferase values for indicated samples. Mutation of miRNA binding site rescues luciferase expression. (**F**) qRT-PCR analysis of miR-138 and TUSC2 from indicated xenograft tumours, transcript abundance is shown as a bar diagram. (**G**) Detection of miR-138 expression in xenograft tumour sections using fluorescent *in situ* hybridization (red signal, upper panel) scale bar-50 µm and immune-histochemical localization of TUSC2 protein (brown stain, bottom panel) scale bar- 100 µm. (**H**) TUSC2 gene expression from TNBC subtype (n = 502) displays an inverse correlation with miR-138 expression as depicted in the Kendall plot. Error bars represent the standard deviation (Student’s *t*-test; *[P < 0.05], **[P < 0.001]).

After demonstrating that miR-138 enhances cell survival in TNBC cell lines, we pursued the molecular mechanisms through which these effects are mediated. We postulated that, as an oncogenic miRNA, miR-138 would post-transcriptionally suppress a suite of downstream messenger RNA transcripts involved in apoptosis and tumour suppression. We compared gene expression profiles from MDA-MB-231 cells transduced with either antagomiR-138 or the scrambled control using a microarray (Fig. 4A). Array data was further validated by qRT-PCR. This survey identified a number of pro-apoptotic and tumour suppressor transcripts with significant differential expression in response to miR-138 knockdown (Fig. 4B). To identify direct targets of miR-138, we performed *in silico* analysis on these RNAs to look for miR-138 binding sites. This computational study revealed the presence of a miR-138 binding site in the *tumour suppressor candidate 2* (*TUSC2*) mRNA (ΔG = −28.9 kcal/mol). *TUSC2*, also known as *FUS1*, is reported in the literature as a candidate tumour suppressor^22^. Unlike the majority of known miRNA target sites which are located in 3′ UTRs, this miR-138 binding site is located within the 5′ untranslated region (5′-UTR) of *TUSC2* where it overlaps with the translation start site (Fig. 4C).

Experimental validation of the predicted miR-138 binding site in *TUSC2* was carried out using a biotin RNA pull-down assay (Fig. 4D). In this assay, a validated miR-138 target, *BLCAP*^23^, was incorporated as a positive control. We obtained significant enrichment of both *TUSC2* and *BLCAP*, but not the negative control *RPLP0*. Data from a chimeric luciferase assay (Fig. 4E) demonstrates that this binding has functional consequences on *TUSC2* expression. We also looked at the effects of miR-138 knockdown on *TUSC2* in xenograft tumour sections (Fig. 4F,G), and observed a clear inverse correlation between miR-138 and *TUSC2* expression. This effect is also corroborated by analysis of miR-138 and TUSC2 levels in TCGA data for TNBC patients (Fig. 4H; Kendall’s rank correlation coefficient = −0.15; p-value = 0.0000112). Taken together, all this confirms that *TUSC2*, a potential tumour suppressor, is a direct target, downregulated by miR-138.

### TUSC2 mimics the effects of miR-138 knockdown

**Figure 5:**
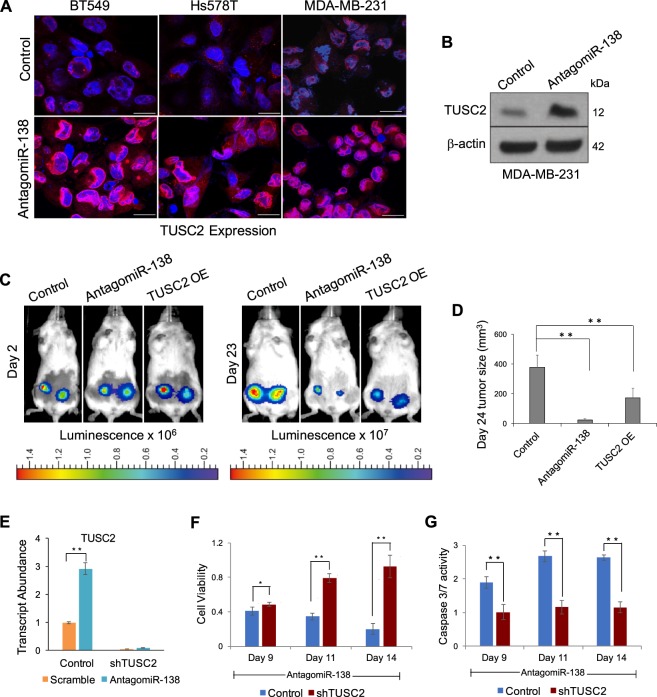
Over-expression of TUSC2 mimics the effects of miR-138 knockdown: (**A**) Immunocytochemistry images of cells following antagomiR-138 transduction scale bar-20 µm. TUSC2 protein is showed in red and nuclei are stained in blue. **B**) Western blot analysis of TUSC2 protein in MDA-MB-231 cell line transduced with indicated lentivirus. β-Actin serves as loading control. (**C**) Representative bioluminescent images of NSG mice implanted with antagomiR-138 expressing or TUSC2 overexpressing or control MDA-MB-231 cells, at indicated time points post engraftment in mammary fat pads. (**D**) Xenograft tumour sizes in mice on day 24 are represented as bar graph from indicated group (n = 10). (**E**) Histogram representing relative transcript abundance of TUSC2 upon miR-138 knockdown in MDA-MB-231 cells, which express control shRNA or shRNA against TUSC2. (**F**) Knockdown of TUSC2 rescues the proliferation defect induced by miR-138 knockdown. Control refers to miR-138 knockdown. (**G**) TUSC2 knockdown decreases caspase 3/7 activity and rescues miR-138 phenotype. Control refers to miR-138 knockdown. Error bars represent the standard deviation (Student’s *t*-test; *[P < 0.05], **[P < 0.01], ***[P < 0.001]).

Having demonstrated that *TUSC2* is a direct target of miR-138, and that miR-138 has pro-survival effects in TNBC cells, we sought further evidence linking the pro-oncogenic function of miR-138 to TUSC2 inhibition. First, we verified that miR-138 knockdown using lentiviral-encoded antagomiR-138 increased TUSC2 in TNBC cell lines. Immunocytochemistry was performed on MDA-MB-231, BT549, and Hs578T using a specific antibody against TUSC2 (Fig. 5A). TUSC2 expression was elevated following miR-138 knockdown relative to controls. Confirming this finding, Western blotting indicated that TUSC2 protein levels are enhanced upon miR-138 knockdown (Fig. 5B and S3D).

To follow up on the effects of TUSC2 as a downstream target of miR-138, we transduced MDA-MB-231 cells with a lentiviral construct encoding *TUSC2*. TUSC2 overexpression in this cell line was validated at both the transcript and protein levels (Fig. S4A). The MDA-MB-231 cell line overexpressing *TUSC2* showed reduced cell number and decreased proliferation compared to controls (Fig. S4B and S4C). For *in vivo* validation of this effect, luciferase-expressing MDA-MB-231 cells were implanted into the mammary fat pads of immunocompromised mice (n = 10) following *ex vivo* transduction with the above constructs. TUSC2 overexpressing cells, along with the miR-138 knockdown cell line and the control cell line, all successfully establish tumours in mice (Fig. 5C). Following at 23 days post-injection, tumours are barely detectable under miR-138 knockdown conditions (Figs 5C, S4D). This observation is consistent with data presented earlier (Fig. 3C). Similarly, implantation of TUSC2 overexpressing cells yields tumours which are significantly smaller than those in the control group (Figs 5C,D, S4D). TUSC2 overexpression therefore substantially mimics the phenotype of miR-138 knockdown and prevents tumour growth. From this finding, we deduce that TUSC2 is a downstream tumour suppressor which is directly repressed by miR-138. It is mainly through TUSC2 repression, that miR-138 exerts its pro-oncogenic effects in TNBC subtype.

Given our findings that TUSC2 overexpression and miR-138 knockdown both inhibit cell proliferation, we aimed to demonstrate whether knockdown of TUSC2 can rescue the reduced cell viability caused by miR-138 knockdown. MDA-MB-231 cells were transduced with a lentiviral construct expressing shRNAs against *TUSC2* (shTUSC2). shRNA expression from this cell line was highly potent; shTUSC2 stables successfully maintained low *TUSC2* transcript expression even under miR-138 knockdown conditions, which would otherwise increase *TUSC2* levels (Fig. 5E). In the absence of miR-138, cell viability was substantially higher in TUSC2 knockdown cells relative to that in control cells expressing antagomiR-138 alone (Fig. 5F). Caspase-3/7 activity, a marker of apoptosis, was also significantly reduced in miR-138 knockdown cells expressing shTUSC2 compared to controls (Fig. 5G). Knock down of TUSC2 therefore rescues the inhibitory effect of miR-138 knockdown on cell proliferation. These results underscore the functional importance of TUSC2 as a tumour suppressor and reveal a path through which miR-138 exerts its pro-oncogenic functions in triple-negative breast cancer.

## Discussion

Triple negative breast cancer is considered incurable with limited therapeutic options, highlighting a dire need for therapeutic targets and predictive biomarkers. Despite systemic therapy, TNBC is an extremely aggressive subtype which is associated with poor prognosis and high mortality rates. Several novel strategies have reached clinical evaluation in patients with TNBC, including targeting poly ADP-ribose polymerase (PARP), epidermal growth factor receptor (EGFR), and Src tyrosine kinase^24^. However, these treatments have not led to significant improvements in the patient outcome^24^. Triple-negative breast tumours are early-onset and more lethal than other breast cancer subtypes due to their site-specific metastatic tendency; TNBCs preferentially spread to the viscera, lungs, and central nervous system^5^.

Dysregulated miRNA expression contributes to initiation and metastasis in a number of cancers^25^. Here we report that miR-138 is highly and specifically expressed in triple-negative breast cancer, making it an ideal biomarker to distinguish TNBC from other breast cancer subtypes. Whilst this concept of subtype-specific miRNA expression profiles in breast cancer is not new^26^, and candidate miRNA biomarkers for TNBC have been proposed^27^, our findings further establish miR-138 as a prognostic biomarker for TNBC. Amongst patients with triple-negative breast tumours, increased miR-138 expression correlates well with poor prognosis, raising the possibility that miR-138 expression level may be informative in the selection of appropriate cancer therapy. It is possible that some of the differences observed in miR-138 expression between TNBC patients may result from sampling of different TNBC subtypes. Unlike luminal and HER2 subtypes, TNBC is heterogenous and may be further subdivided into molecular subtypes based on mRNA profiling^28^. We postulate an effect of TNBC subtype on patient survival, which we predict would directly correlate with subtype-specific miR-138 expression.

The use of targeted treatments in the management of hormone receptor-expressing and/or HER2-positive breast cancers has revolutionized the treatment of these diseases. Hormone therapy, such as the use of Tamoxifen, may be used to block signalling through the oestrogen receptor. Trastuzumab, a humanized monoclonal antibody, is used to specifically target the HER2 receptor^26^. Such advances have proven elusive in the context of TNBC due to the absence of hormone receptor and HER2 expression. There is a pressing lack of targeted treatment for triple-negative breast cancer, which represents an increasingly feared diagnosis amongst breast cancer patients. Our discovery that miR-138 is functionally involved as an oncogenic driver in TNBC raises the possibility that, in addition to its utility as a biomarker, miR-138 may soon represent a druggable target for oligonucleotide-based therapeutics. It is also likely that our discovery of a pro-survival function for miR-138 applies to other malignancies. Indeed, we have previously demonstrated that miR-138 is pro-oncogenic in malignant glioma, where it promotes the survival of glioma stem cells in a manner similar to that observed in TNBC cells^23^. These findings present the tantalizing prospect that a targeted therapeutic strategy aimed at reducing miR-138 expression may be deployed in the management of multiple different cancers. With the development of novel targeted strategies to efficiently deliver oligonucleotides into tumour cells, we envision the potential value of using antagomiR-138 to treat this deadly cancer.

Potential therapeutic targets for TNBC need not be restricted to miR-138. Our identification of downstream miR-138 targets circumvents current problems related to directly targeting miR-138. TUSC2, a tumour suppressor which is directly targeted by miR-138 in the context of triple-negative breast carcinoma, performs similar anti-tumour functions in other cancers^22^. Loss of TUSC2 expression has been observed in lung carcinoma due to either a 3p21.3 deletion^29^ or post-transcriptional repression by microRNAs^30,31^. Strategies aimed at restoring TUSC2 are currently in clinical trials^22^. If successful, these therapeutic regimes may be used to complement current clinical practices in treating TNBC. Furthermore, candidate therapeutic targets involved in the miR-138 regulatory network are by no means restricted to miR-138 and TUSC2. Our microarray data reveal a number of other genes related to cell growth and survival which are altered by miR-138, and the partial rescue phenotype observed for shTUSC2 expression in a miR-138 knockdown background suggests that other factors contribute to the pro-survival effect of miR-138. We therefore postulate that additional components of the miR-138 regulatory network may operate in the context of TNBC, which are worthy of further study.

Thus, gaining an understanding of miRNA dysregulation in cancer, coupled with the development of RNA-targeted therapeutic approaches, opens up the potential to combat previously undruggable cancers including triple-negative breast cancer (TNBC).

## Materials and Methods

### Cell culture

Breast cancer cell lines MDA-MB-231, MCF-7, SKBr3 and Hs578T were cultured in Dulbecco’s Modified Eagle’s Medium (DMEM) (#11960-044, Gibco) with 10% FBS, 1% glutamine, and penicillin/streptomycin. BT549 and T47D cells were cultured in RPMI-1640 Medium (Gibco) with 10% FBS, 1% glutamine, and penicillin/streptomycin.

#### miRNA *in situ* hybridization

Five micron sections were processed and boiled in pre-treatment solution (Panomics), washed in PBS, followed by protease (Panomics) treatment at 37 °C. Sections were incubated with LNA probes [5′-DIG labelled LNA probes specific for miR-138 or scrambled probe with no homology to known vertebrate miRNAs (Exiqon)] in hybridization buffer (Roche) at 51 °C for 4 hours. Adapted from Pascale *et al*.^32^. Following stringent washing with 5×, 1x and 0.3x SSC buffers, sections were blocked with 10% Goat serum and further incubated with anti-DIG alkaline phosphatase (Roche) overnight at 4 °C. Sections were washed in PBS-T (0.1%) and miRNA bound LNA probes were detected by Fast red substrate (Panomics). After counterstaining with DAPI, slides were mounted using FluorSave (Merck). Image acquisition was performed using the Olympus FluoView FV1000 using TRITC filter. Adapted from Sundaram *et al*.^33^. Breast cancer tissue arrays (BR1503e) were obtained from US Biomax.

#### *In-vivo* tumour inhibition assays

Six- to eight-week-old female NOD-scid IL2Rg^null^ inbred mice were obtained from Jackson Laboratories, Bar Harbor, ME, USA and housed in a specific pathogen-free animal facility. The animals were fed with irradiated mouse chow and autoclaved reverse osmosis treated water. All the animal procedures were performed in accordance with a protocol approved by the Agency for Science Technology and Research (A*STAR) Institutional Animal Care and Usage Committee (IACUC #171231). MDA-MB-231 cells constitutively expressing luciferase, transduced with lentiviruses expressing control shRNA or shRNAs against miR-138 or over-expressing TUSC2 were harvested with trypsin/EDTA. Cells were washed and re-suspended in growth media. Eight-week old female mice were injected unilaterally with 2.5 × 10^6^ cells in 200 µL of 50∶50 Matrigel/Collagen I into the fourth abdominal fat pad by subcutaneous injection at the base of the nipple. Survival and successful injection of the cells was monitored by detection of bioluminescence at the site of injection after 24 hours using IVIS Spectrum *in-vivo* Imaging System (Xenogen, Perkin Elmer, MA, USA). In each imaging session, a total of 150 mg of Luciferin per kg body weight was administered into the peritoneal cavity. Mice were imaged 9 minutes after Luciferin injection to ensure consistent photon flux. The bioluminescent signal was expressed in photons per second and displayed as an intensity map. The image display was adjusted to provide optimal contrast and resolution in the image without affecting quantitation. Following acquisition, all images were normalized to units of average efficiency, displayed in the same scale of luminescence intensity, and analyzed using the Living Image 4 software (Xenogen, Perkin Elmer, MA, USA). Tumour growth was also monitored externally using vernier calipers. Luminescence from the cells was measured in the fat pads using a region of interest tool. 32 days post injection mice were euthanized, tumours were harvested, and a portion of them was homogenized in TRIzol® for RNA isolation and the remaining was fixed and processed into FFPE blocks. Adapted from Sundaram *et al*.^34^.

#### Western blotting

Cells were directly lysed from 6-well plates by scraping in RIPA buffer. After clarifying the lysate by centrifuging at 13,000 rpm at 4 °C, total protein was quantitated by Bradford Protein Assay (Bio-Rad). Equal amount of (30 μg) total protein was subjected to SDS-PAGE followed by Western blotting with standard protocols. After primary and secondary antibody incubation and washing, proteins were visualized by ECL western detection reagent (Millipore Crescendo). Band intensities were quantified using ImageJ software (National Institute of Health, USA). Signal intensities were normalized to their appropriate loading controls. Adapted from Sundaram *et al*.^34^.

#### Antibodies

Antibodies used in this study are as follows. Rabbit anti-cleaved PARP (ab32561) and rabbit anti-TUSC2 (ab70182) were from Abcam. Rabbit anti-PARP (9542), rabbit anti-caspase-3 (9662), rabbit anti-cleaved caspse-3 (9661) and rabbit anti-β-Actin (5125) were from Cell Signalling Technology. Rabbit anti-phospho-Histone H3 (06–570) was from Merck and rabbit anti-Ki-67 (NB600-1252) was from Novus Bio. Donkey anti-rabbit Alexa Fluor 555 were from Molecular Probes.

#### Cell proliferation and viability assay

To assess the effect of miR-138 knock down or TUSC2 knockdown on cell proliferation, cells transduced with control shRNAs or shRNAs against miR-138/TUSC2 or both were seeded at a density of 150 cells per well in 96 wells in complete medium. Cell proliferation was measured at different days post seeding using CellTiter-Glo® Luminescent “Cell viability assay” (Promega) as per manufacturer’s instructions using a luminometer. Experiments were performed in two biological replicates with at least eight technical replicates per condition. Adapted from Sundaram *et al*.^34^.

#### Caspase 3/7 assay

To assess the effect of miR-138 knockdown or TUSC2 knockdown on caspase 3/7 activity, cells transduced with control shRNAs or shRNAs against miR-138/TUSC2 or both were seeded at a density of 150 cells per well in 96 wells in complete medium. Cell proliferation was measured at different days post seeding using Caspase-Glo® 3/7 Assay Systems (Promega) as per manufacturer’s instructions using a luminometer. Experiments were performed in two biological replicates with at least eight technical replicates per condition. Adapted from Pascale *et al*.^32^.

#### Soft agar assay

Single-cell suspension of 2 × 10^3^ cells (MDA-MB-231 and MCF-7) was plated in medium containing 0.3% noble agar (Difco) seeded in 6-well plates containing 1% noble agar. Cells were cultured for 2–3 weeks with growth medium supplementation. Lentiviral-transduced cells were subjected to puromycin selection. Cells were incubated with 1 mg/ml 3-(4, 5-dimethylthiazol-2-yl)-2, 5-diphenyltetrazolium bromide (Sigma-Aldrich), and colonies were counted using MATLAB software. Adapted from Chan *et al*.^23^.

#### Cell-Cycle analysis

Lentivirus-transduced cells (MDA-MB-231, BT549 and Hs578T) were plated at a density of 100,000 cells/well, labelled with 10 mM EdU for 5 hr prior to harvesting, and processed using the Click-iT EdU Alexa Fluor 647 Flow Cytometry assay (Invitrogen). Cell nuclei were counterstained with 25 mg/ml propidium iodide (Sigma-Aldrich). Samples were subjected to EdU incorporation analysis on a BD FACS caliber (Becton Dickinson). Data were analyzed using WINMDI 2.9 software Adapted from Chan *et al*.^23^.

#### Immunohistochemistry

Five-micron tissue sections were mounted on polylysine-coated glass slides (Thermo Scientific). Sections were washed in xylene and rehydrated using a graded ethanol series finishing in phosphate buffered saline (PBS). Endogenous peroxidase activity was quenched by immersing the slides in 3% hydrogen peroxide for 30 minutes. Antigen retrieval was performed using programmable pressure cooker with “target retrieval solution”, pH 6.0 (Dako). Non-specific reactivity in the tissues was blocked by incubation in 10% goat serum in PBS before incubating with the primary antibody at room temperature. Unbound primary antibodies were removed before incubation with species matched secondary HRP-labelled polymer antibodies (Dako). Chromogen 3, 3′-diaminobenzidine (Dako) was used as substrate for colour development. Slides were counterstained with hematoxylin before dehydration and mounted with DPX (Sigma). For fluorescent immunodetection, species-specific secondary antibodies conjugated to Alexa 488/555 were used instead of HRP-labelled polymer antibodies. Sections were washed, counterstained with DAPI (100 ng/ml) and mounted using FluorSave (Calbiochem) mounting medium. For experiments where goat primary antibodies were used, 5% BSA in PBS was substituted for 10% goat serum. Images were acquired on a Zeiss Axioimager microscope (for bright field imaging) or on Olympus FluoView FV1000 (for fluorescent antibody detection). Adapted from Sundaram *et al*.^34^.

#### Quantitative RT-PCR

RNA was isolated from cells using the miRCURY RNA Isolation Kit (Exiqon). cDNA was synthesized from small non-coding RNA using the miRNA RT assay (TaqMan). Expression levels of miRNA were measured on a 7900 fast RT-PCR system (Applied biosystems) in triplicates using 10 ng/µl cDNA and TaqMan probes specific for miR-138. U6 probe was used as an endogenous control. The ΔΔCt method was applied to determine the transcript abundance. For cDNA preparation from total mRNA, SuperScript III Reverse Transcriptase was used. Quantitative Real Time PCR (qRT-PCR) analyses were performed using primers that amplify a coding region of the TUSC2 gene. qRT-PCR was performed in triplicates with SYBR™ Green master mix (Applied Biosystems), 0.2 uM primers and 10 ng/µl cDNA. Adapted from Pascale *et al*.^32^.

#### Preparation of Lentiviral Stocks and Transduction

Stable expression of antagomiRs was carried out using miRZip, a lentiviral expression vector (System Biosciences). Mature functional antagomiR-138 sequence is CGGCCTGATTCACAACACCAGCT. The H1 expression cassette provides constitutive RNA polymerase III-dependent transcription of antagomiR transcripts. CMV promoter supports expression of copGFP (fluorescent reporter) and puromycin-N-acetyl transferase (drug-selectable marker) for detection and selection of transduced cells, respectively. Lentivirus expressing luciferase under human PGK promoter was obtained from Addgene. Adapted from Chan *et al*.^23^. Third-generation lentiviruses were produced in Lenti-X 293 T (Clontech) with packaging mix consisting of three constructs, pMDLg/pRRE (#12251), pRSV-Rev (#12253), and pMD2.G (#12259), from Addgene. Supercoiled DNA constructs were prepared using Plasmid Maxi Kit (Omega bio-tek).

#### Microarray Analysis for Determination of Gene Expression Profile

Total RNA (500 ng) from three replicates of TNBC cell line, MDA-MB-231 transduced with antagomiR-138 or scrambled control was converted to biotinylated cRNA using Target Amp Nano-g Biotin-aRNA labelling kit (Epicenter) and isolated using QIAGEN columns. cRNA was hybridized on HumanWG-6 v3.0 array (Illumina). Normalized data were analysed with Illumina Bead Studio, and analysis was performed on PARTEK platform. Adapted from Chan *et al*.^23^. GEO accession number is GSE110659.

#### RNA immunoprecipitation using Biotinylated mimics

HEK293T cells (1 × 10^6^) were transfected in triplicate with biotinylated mimics of miR-138 or scrambled (Dharmacon) using RNAi max transfection reagent as per manufacturer’s protocol. Twenty-four hours later, the cells from 3 wells were pelleted at 500 × g. After washing twice with PBS, cell pellets were re-suspended in 0.7 ml lysis buffer (20 mM Tris (pH 7.5), 100 mM KCl, 5 mM MgCl_2_, 0.3% NP-40, 50 U of RNase OUT (Invitrogen), complete mini-protease inhibitor cocktail (Roche Applied Science), and incubated on ice for 5 min. The cytoplasmic lysate was isolated by centrifugation at 10,000 × g for 10 min. Streptavidin-coated magnetic beads (Invitrogen) were blocked for 2 hr at 4 °C in lysis buffer containing 1 mg/ml yeast tRNA and 1 mg/ml BSA (Ambion) and washed twice with 1 ml lysis buffer. Cytoplasmic lysate was added to the beads and incubated for 4 h at 4 °C before the beads were washed five times with 1 ml lysis buffer. RNA bound to the beads (pull-down RNA) or from 10% of the extract (input RNA), was isolated using Trizol LS reagent (Invitrogen). The level of mRNA in the miR-138 or scrambled control pull-down was quantified by qRT-PCR. The enrichment ratio of the control-normalized pull-down RNA to the control-normalized input levels was then calculated. Adapted from Lal *et al*.^35^.

#### Gene expression analysis of hsa-miR-138 using TCGA breast cancer dataset

Level 3 miRNA gene expression data from TCGA was downloaded. Expression files category “miRbase20 isoform quantification” was chosen to obtain the expression of mature miR-138. Short reads mapped to any sequence within the mature form of miR-138 (5′-AGCUGGUGUUGUGAAUCAGGCCG-3′) were counted. Total reads mapped to 29 reported observed isoforms were summed to represent the expression of mature miR-138. The reads counts were then normalized by the library depth. Expression profiles of miR-138 in breast cancer intrinsic sub-types along with healthy samples were compared using Wilcoxon test. Information of intrinsic subtypes was obtained from The Cancer Genome Atlas Network 2012^36^.

#### Survival analysis of miR-138 using public survival tool

The prognostic significance of miR-138 was assessed using breast cancer patient’s follow-up data provided by miRpower tool (Lánczky 2016). The association of overall survival with miR-138 expression profile was assessed in all samples and within triple negative subtype samples (TNBC) only. Patient population was split by upper quartile option.

#### Statistical analysis

Values are reported as the mean ± the standard deviation. Statistical significance between 2 samples was determined with two-tailed Student’s *t*-test or one-way analysis of variance when comparing multiple groups. Adapted from Sundaram *et al*.^34^.

## Supplementary information

Supplementary information
