# Supplementary material for: MicroRNA-138 is a Prognostic Biomarker for Triple-Negative Breast Cancer and Promotes Tumorigenesis via TUSC2 repression
Source: Sci Rep. 2019 Sep 3;9:12718. doi: 10.1038/s41598-019-49155-4 (PMC6722084; doi:10.1038/s41598-019-49155-4)
Supplement: Supplementary file 1 — Supplementary information [file 41598_2019_49155_MOESM1_ESM.pdf]

MicroRNA-138 is a Prognostic Biomarker for Triple-Negative Breast Cancer and Promotes Tumorigenesis via TUSC2 repression

Srikanth Nama[1, #], Manish Muhuri[2, #], Federica Di Pascale[1], Shan Quah[1], Luay Aswad[5,6], Melissa Fullwood[5,6] and Prabha Sampath[1, 3, 4, *]

Supplementary Information:

Supplementary Figures and Figure Legends:

Figure. S1

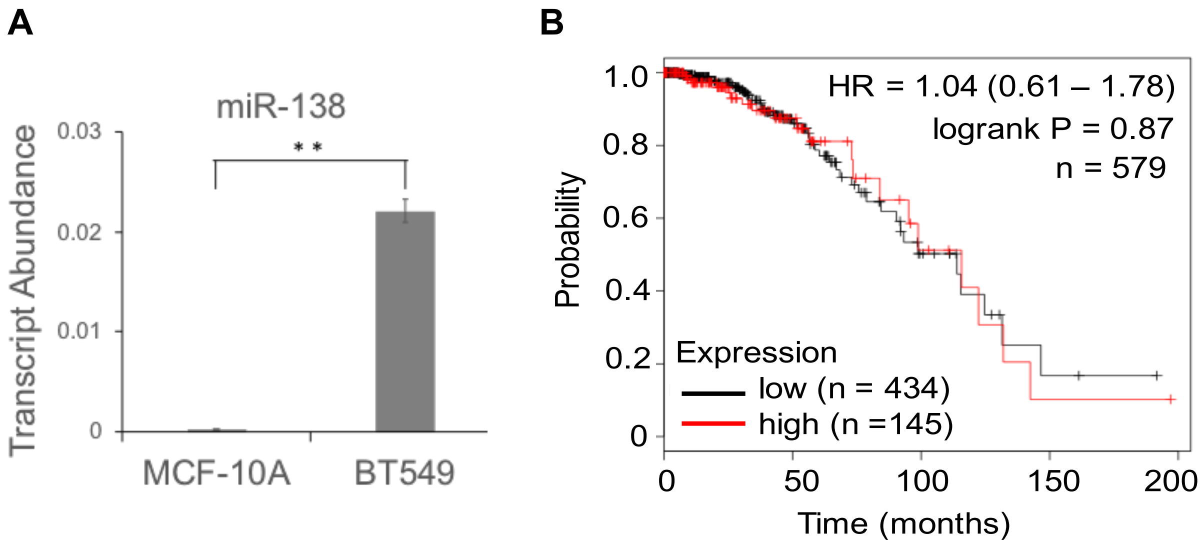

Figure S1: Expression profile of miR-138: A) Bar graph depicts expression of miR-138 in normal and breast cancer cell line. **B)** Kaplan-Meier survival curves with statistical significance by log-rank test of two groups, in which breast cancer patients were grouped as low miR-138 expression (n = 434) and high miR-138 expression (n = 145). Error bars represent the standard deviation (Student's *t*-test; **[P < 0.001]).

Figure. S2

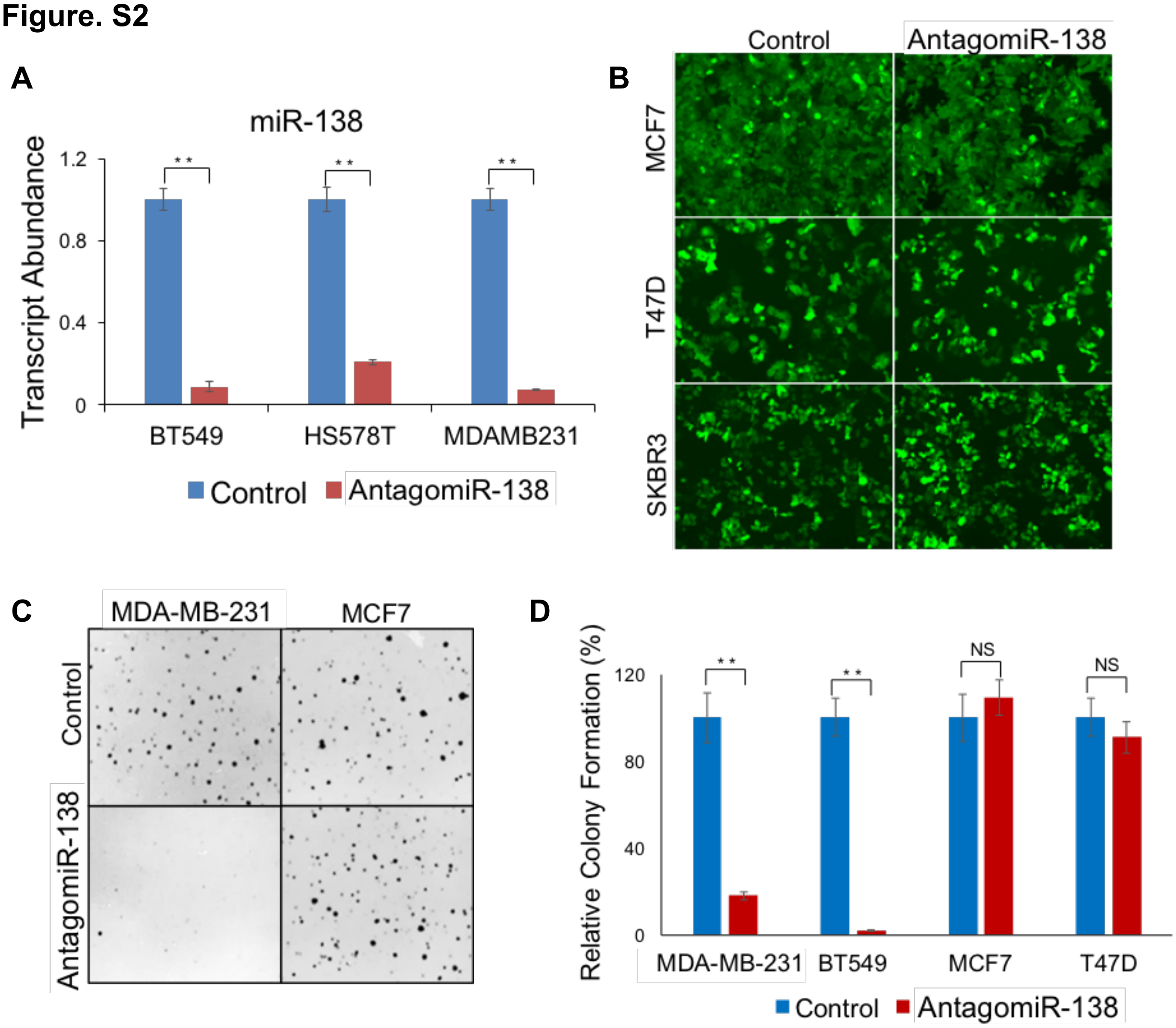

Figure S2: Knockdown of miR-138 leads to colony suppression A] Bar graph depicting transcript abundance of miR-138 in 3 cell lines derived from TNBC subtype transduced with indicated lentivirus expressing antagomiR-138 or scrambled control. **B]** Fluorescent images from cells (3 cell lines derived from HR+ve subtype) transduced with indicated lentivirus expressing antagomiR-138 or scrambled control. **C]** Images represent colony suppression analysis of MDA-MB-231 and MCF-7 cells transduced with indicated lentiviruses. **D]** Bar graph illustrating relative colony formation in indicated cell lines transduced either with control (scramble) or with antagomiR-138. Error bars represent the standard deviation (Student's *t*-test; **[P < 0.001]).

Figure. S3

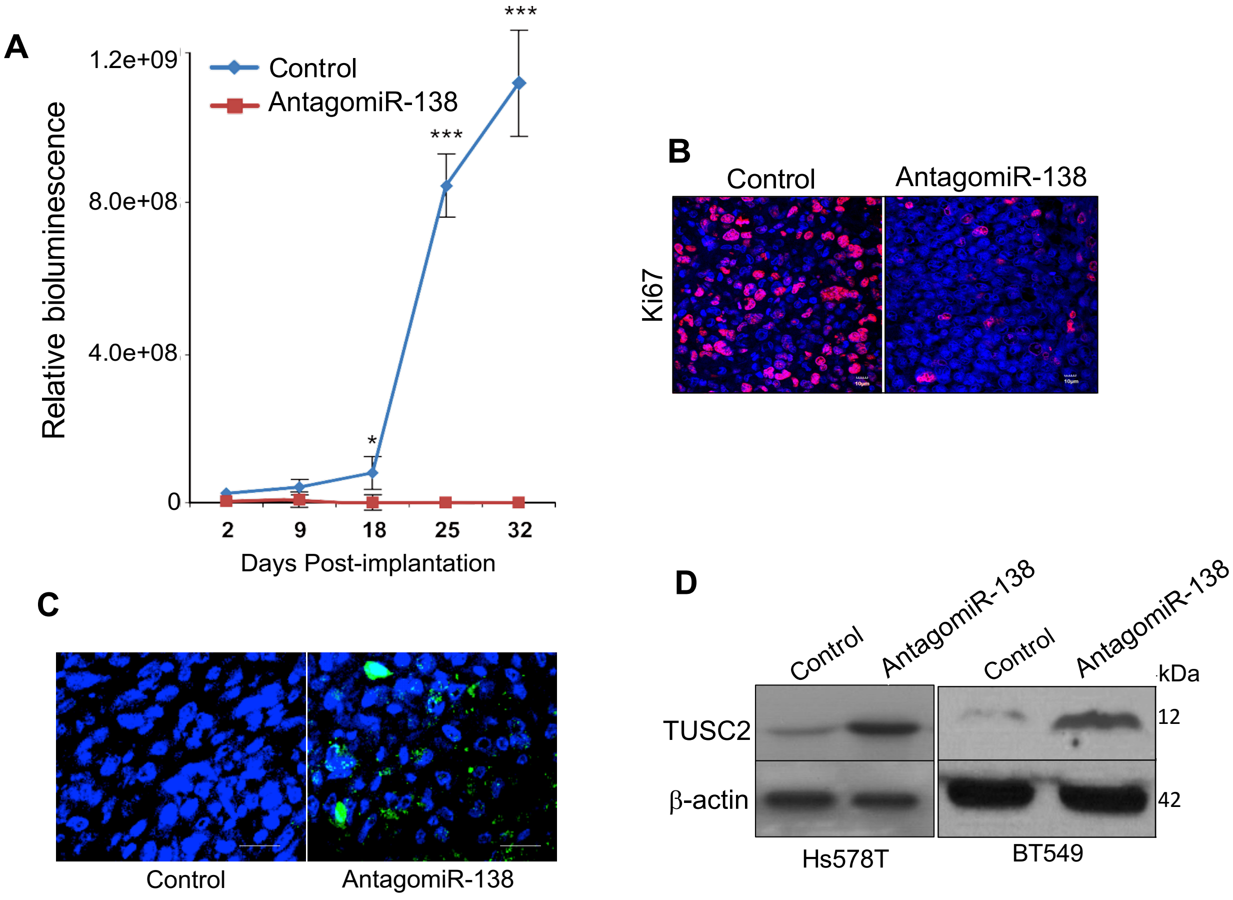

Figure S3: MicroRNA-138 is a pro-survival oncomiR: A] Line graph illustrating real time quantification of xenograft tumour growth in terms of bioluminescence (n = 10) at indicated time points. Note that antagomiR-138 transduced cells showed decreased bioluminescence compared to control transduced cells. **B]** Knockdown of miR-138 decreases tumor cell proliferation as seen by Ki-67 marker in xenografts tumor sections. Scale bar, 10 µm. **C]** Knockdown of miR-138 leads to apoptotic cell death as observed by caspase expression in xenografts tumor sections **D]** Western blot analysis of TUSC2 protein from BT549 and MDA-MB-231 cells transduced with indicated lentivirus. β-Actin serves as loading control.

Figure. S4

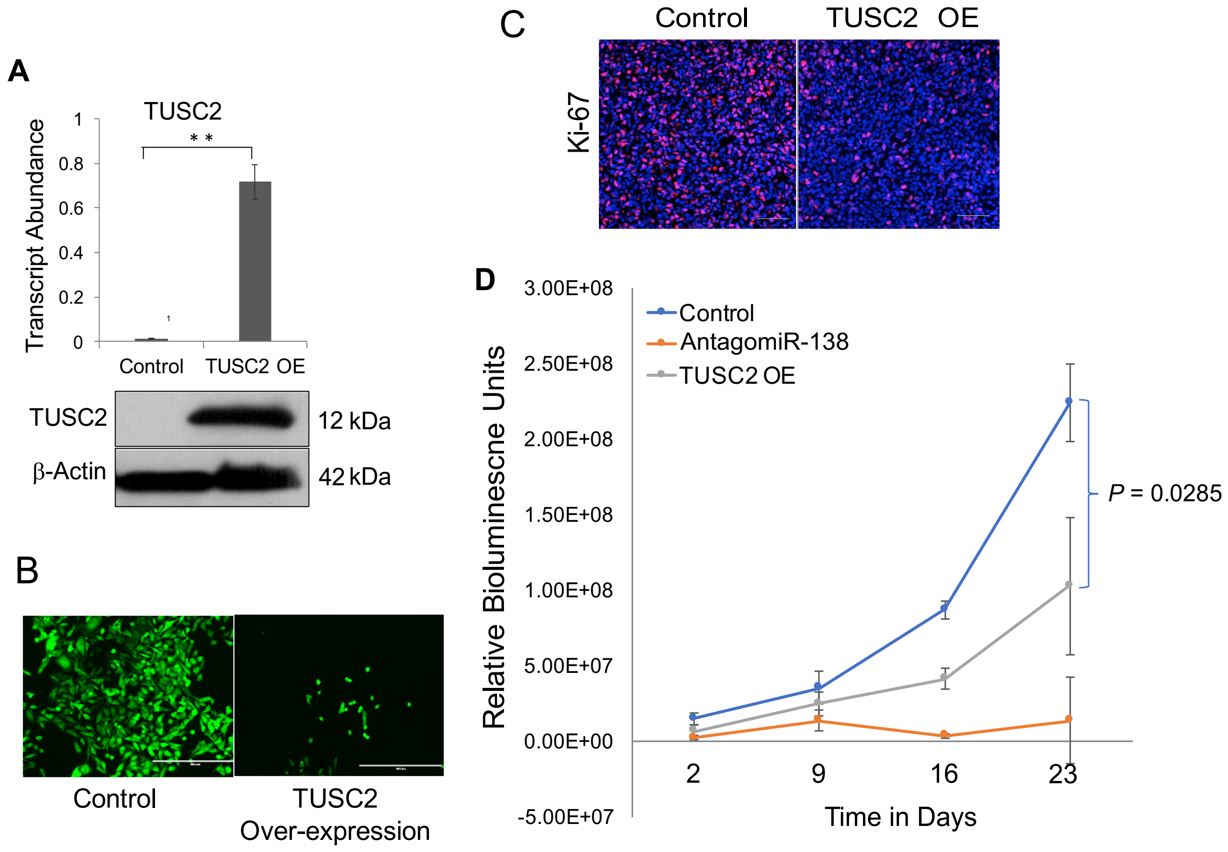

Figure S4: Over-expression of TUSC2 in MDA-MB-231: A] Transduction of MDA-MB-231 cells with lentivirus encoding TUSC2 results in high levels of TUSC2 transcript and protein. **B]** Images of MDA-MB-231 cells transduced either with empty vector or with TUSC2 over-expression vectors at day-7 post transduction. Scale bar, 100 μm. **C]** Over-expression of TUSC2 decreases tumor cell proliferation as seen by Ki-67 marker in xenografts tumor sections. Scale bar, 10 μm. **D]** Line graph representing bioluminescence data from mammary fat pad tumors in NSG mice implanted with indicated samples. TUSC2 over expression and antagomiR-138 transduced cells showed significant tumour growth inhibition compared to control cells.

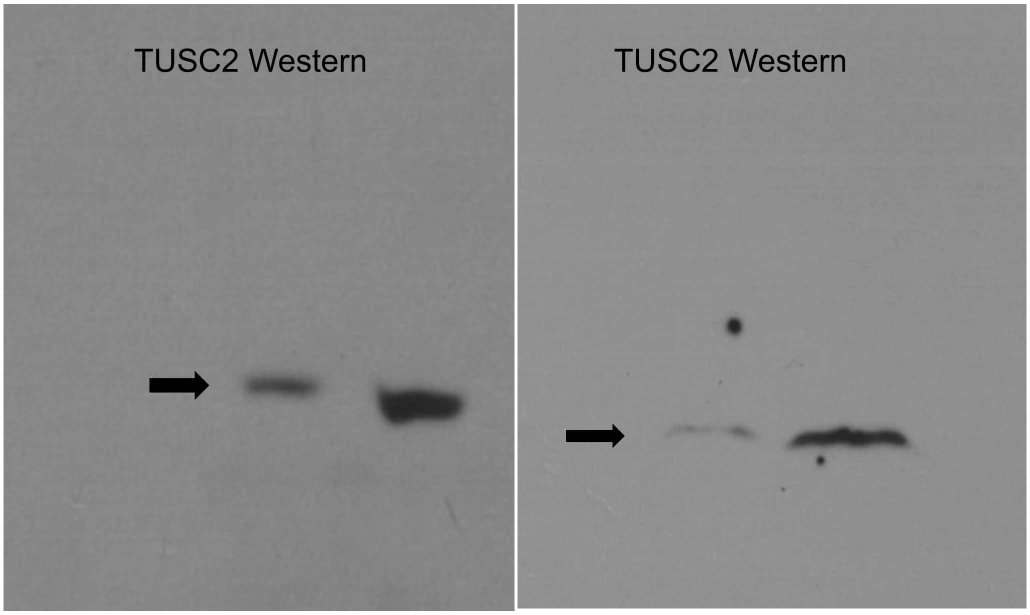

Full gel picture of Western blot for TUSC2

PRIMER NAME	SEQUENCE
RPLP0_F1	CAGATTGGCTACCCAACTGTT
RPLP0_R1	GGGAAGGTGTAATCCGTCTCC
AURKA_F1	GGGTGGTCAGTACATGCTC
AURKA_R1	GCATCCGACCTTCAATCATTTC
HIF1A_F1	CAGCAGCCAGACGATCATGCA
HIF1A_R1	TGGTCAGCTGTGGTAATCCACTTTCA
LASP1_F1	TCACCACATCCCGACCAG
LASP1_R1	CGGCGCTGTAGTCATACACC
BLCAP_F1	GTCGGTGGCGAGCTGAGG
BLCAP_R1	CACCAAGGCAGCAGGGATC
GADD45A_F1	CCCTGATCCAGGCGTTTTG
GADD45A_R1	GATCCATGTAGCGACTTTCC
MXD1_F1	AGCCGTTCACCAAATCGACC
MXD1_R1	CTCGTCAGAGTCGCTCAC
PANX2_F1	CCAAGAACTTCGCAGAGGAAC
PANX2_R1	GGGCAGGAACTTGTGCTC
TUSC2_F1	ATGATGAGGATGGGGATCTG
TUSC2-_R1	GAGGATCACAGGGAAATCCAC
TXNIP_F1	AGTGCAAACAGACTTCGGAG
TXNIP_R1	TTTGTCTCTTGAGTTGGCTGG
miR138_BINDINGSITE_WT	GGCGGCCCTGACATGGGCGCCAGCGGG
miR138_BINDINGSITE_MUT	GGCCAGGCTGACATGGGATGGTCAGGG
TUSC2_shRNA	GGTTTGTAAATAGTCCATG
